# Editorial: Persistence of measurement problems in psychological research

**DOI:** 10.3389/fpsyg.2023.1132185

**Published:** 2023-01-23

**Authors:** Scott T. Meier

**Affiliations:** Department of Counseling, School, and Educational Psychology, University at Buffalo, Buffalo, NY, United States

**Keywords:** measurement, psychology, replication, scientific crises, testing

## Introduction

As we observed in the announcement of this Research Topic, reviews of the history of science suggest that new measurement approaches drive scientific development (Kuhn, 1970; Cone and Foster, 1991; Tryon, 1991; Meier, 1994, 2008). Tryon (1991) wrote that scientific progress results from a measurement method's capacity to correct and extend human senses into new domains and provide new data that transforms theory. Small improvements in measurement can accumulate and result in significant observational advances (Meier, 1994, 2008). Despite decades of recognition of measurement problems in psychology and related fields, contemporary scholars continue to raise alarms about the state of psychological measurement. Longstanding problems such as the validity of self-reports and inconsistencies in test scores across sources (e.g., ratings of children by parents, teachers, and children) will recur until the field finds effective solutions (Benjamin and Baker, 2009; Lilienfeld, 2017). Historical references provide evidence that psychology's measurement problems are not random occurrences, but periodic problems that appear, fade away without resolution, and are later rediscovered (Meier, 1994, 2008; Lilienfeld, 2017).

## Critique of current methods

Lilienfeld and Strother (2020, p. 281) wrote that “many researchers pay little heed to the psychometric properties of their measures, cavalierly neglecting them, or taking them for granted.” They provided examples of four irrational beliefs about measurement that contribute to the replication crisis in psychology: (a) the name of a measure reflects its content, (b) reliability is not a major concern for laboratory measures, (c) large sample sizes are unnecessary when data are difficult to collect, and (d) construct validity can be adequately assessed by estimates of convergent validity alone. Similarly, Flake and Fried (2020) placed the key source of problems as what they termed a *measurement schmeasurement* attitude whereby researchers and other users of psychological tests sidestep measurement problems by ignoring them.

Another indicator of the neglect of measurement is a tendency for researchers to operationalize a construct through a single test rather than expend the resources necessary to conduct a thorough construct explication (cf. Scheel et al., 2021). Edison's attempt to find a proper filament for the light bulb offers an appropriate metaphor here. When Edison invented the light bulb, he reportedly tested 3,000 of types of materials to identify filaments that generated light but minimized heat (Palermo, 2017). In contrast, to believe that a single iteration of a psychological test, often developed in the early stages of research in a domain, represents the best explication of any single construct appears highly unlikely.

Nevertheless, examples abound of psychological tests and operations that have been adopted as the standard, default method in many areas of research with minimal discussion of potential alternatives. Bianchi et al. (2015) found that in a review of measures employed in research on occupational stress and burnout, the self-report Maslach Burnout Inventory (MBI; Maslach and Jackson, 1981) was employed in ~80% of review studies. Similarly, in the domain of working alliance research in psychotherapy, the Working Alliance Inventory (WAI; Horvath and Greenberg, 1989) has been employed in ~70% of studies (Flückiger et al., 2018; Meier and Feeley, 2021). This constitutes both a mono-operation bias and mono-method bias in that research findings will be influenced by use of a single test employing a single method (Campbell and Fiske, 1959).

Finally, in the quest to find statistical significance (Nosek et al., 2013; Ledgerwood, 2014), quantitative researchers typically conduct a power analysis focused on sample size and expected effect size, and then adjust the former to increase power to detect an effect (Cohen, 1992; Houle et al., 2005; Drummond and Vowler, 2012). The strategy to increase sample size increases the likelihood of finding a statistically significant finding, which increases the odds of publication, but often results in the detection of a small to moderate effects (Lipsey, 1990) that are difficult to replicate. At the analysis end of a study, researchers employ advanced statistical methods such as structural equation modeling and item response theory that provide the veneer of scientific sophistication but whose results largely depend upon the quality of the data produced during measurement (Cone and Foster, 1991).

A parallel line of thinking is evident in attempts to identify questionable research practices (QRPs) related to statistical procedures that may potentially create problems for subsequent attempts at replication. Discussing QRPs, Ulrich and Miller (2020) proposed that the base rate of true effects strongly influences replication rate in scientific results. Their central thesis is that “low power within a research area reduces replicability for purely statistical reasons, because it reduces the ratio of true positives to false positives” (p. 2). From this perspective, strategies such as data peeking and selective reporting have little effect on replication rate. They conclude that “low base rates of true effects—not too-large *a* levels, too-low power, or p-hacking—are most likely to be the major causes of poor replicability, so researchers concerned about replicability should pay special attention to the issue of base rates” (p. 18). If studies of psychological effects evidence low base rates, then careful development of psychological tests able to detect small to moderate effects would seem to be of paramount importance.

## Alternatives to current practices

Method effects (MEs) refer to the observation that scores on every quantitative variable, index, and measure at least partially reflect the methodology employed to collect data. Cote and Buckley's (1987) research (Williams et al., 1989) concluded that about 25% of variance in scores on a typical measure results from sample and measurement characteristics. Even seemingly minor methodological conditions can influence results. Studies have found, for example, that (a) the gender of a researcher present in an experimental setting could influence the behavioral performance of rats and mice and (b) a priming study's results were unintentionally influenced by the fact that the researcher who packaged materials for the priming or control groups was also the individual who handed the materials to all participants (Brown et al., 2014).

Historically, one of the goals of the test development process was to reduce or eliminate MEs in psychological measurement. A ceiling effect in a set of test scores, for example, should not be present because items or scales with skewed scores are typically identified and eliminated during the item analysis procedure. MEs can provide clues about where test developers should re-examine construct explication, the process of connecting theoretical constructs to observed behaviors (Torgerson, 1958). When MEs are present, a problem has occurred in explication, and exploration of the problem provides an opportunity to deepen substantive knowledge and improve the power of measurement devices. Construct explication consists of four resource-intensive steps.

1. Review and/or develop substantive theory related to the construct(s).
2. Review and/or develop methodological theory related to the construct(s).
3. Employ the results of one and two to create appropriate measure(s).
4. Repeat the process in a program of research to improve the power of developed measures to detect effects of interest.

Step 2 is often problematic in contemporary psychological study: Researchers may minimize methodological considerations, hence, *measurement schmeasurement* (Flake and Fried, 2020). In any study, methodological decisions must be made regarding who will be measured (sampling), how the data should be observed (test characteristics), and how the data will be employed (test purpose). In much contemporary research, however, methodology has become detached from theories about the construct, with (a) convenience sampling, (b) self-report as the default method of data collection, and (c) coefficient alpha, factor analysis, and correlational procedures as the default analyses for evaluating the quality of item response data (Maul, 2017).

The major paradigm for psychological testing historically has been to select persons for entrance to educational, business, and military settings on the basis of individuals' measured traits. Consequently, test developers have favored trait-based items and tasks designed to discriminate among individuals and predict future performance (Dawis, 1987; Danziger, 1990) and have sought items that maximize stability over time and detection of individual differences. For other testing purposes, however, this paradigm can reduce power.

Even when the goal of a test is to detect intervention effects (Lipsey, 1983, 1990; Tryon, 1991; Meier, 1994) test developers and users may default to selection-based testing procedures. Stinchfield et al. (2007) created the Gambling Treatment Outcome Monitoring System (GAMTOMS), a measure intended to assess changes in gambling behaviors following treatment. Using 286 participants (including 237 gambling treatment clients) in 2 studies, Stinchfield et al. provided evidence for the GAMTOMS' internal consistency, 1-week test-retest reliability, content validity, convergent validity, discriminant validity, predictive validity, and construct validity. Change-sensitivity would appear to be a critical criterion for evaluating the GAMTOMS' intended purpose, that is, the power to detect change in gambling behaviors following intervention. With the exception of a single item examining stages of change, no analyses evaluated whether GAMTOMS' items or scores could detect change over time or in response to an intervention.

## Conclusion

Hirsch (2009) provided a historical perspective regarding how scientists in any scientific domain make progress in measurement.

A young discipline is bound to move first through the data it can gather most easily. And as it does, it also defines more exactly what it must measure to test its theories. As the low-hanging fruit vanish, and the most precious of fruits are spotted high above, bigger investments in harvesting equipment become necessary.

Psychology has harvested its low-hanging fruit, primarily through self-report, interview, and experimental methodologies that simply operationalize (rather than evaluate) constructs. We challenge researchers and test developers to (a) evaluate a measurement issue in every study you conduct, (b) build a knowledge base and accompanying questions about how method affects findings with the constructs you research, and (c) implement a new measurement procedure during pilot studies. Noteworthy examples in this regard can found in (a) Charamut's et al. description of the trait, context, and source effects in measurement of youth mental health, (b) Dohrenwend's (2006) discussion of intracategory variability on stress self-report measures and a possible solution with narrative rating scales, and (c) Tryon's (1991) analysis of how trait and state effects can be separated and detected in a single dataset.

As a field we must systematically step back and think more deeply about how to measure and better detect the effects of psychological phenomena of interest. Failure to pursue new directions means that research crises such as the replication problem will recur. The studies in this Research Topic, summarized below, offer examples of innovative possibilities in psychological measurement.

## Summary of Research Topic manuscripts

### Multi-informant reports

Charamut et al. observed that assessment of youth mental health problems typically involves data collection from multiple informants that can vary substantially. One explanation is situational specificity: Children and adolescents vary in the situations where they display problem behaviors, and observers such as teachers and parents vary in where they observe these behaviors. Charamut et al. presents a sophisticated evaluation of Kraemer's et al. (2003) Satellite Model that consists of the context in which an informant observes the youth undergoing evaluation as well as the source of data (e.g., self vs. other). Users of this approach select informants who vary in their contexts and perspectives, thus allowing for a third component (i.e., trait) to reflect common variance, aspects that generalize across informants' contexts and perspectives. Thus, the Satellite Model examines both common variance (i.e., trait) and domain-relevant unique variance (i.e., context and perspectives).

Charamut's et al. research employed 134 clinical and community adolescents ages 14–15 and their parents who completed six parallel measures of adolescent mental health. The measures assessed social anxiety, social phobia, fear of evaluation, work and social adjustments, and depression. Adolescents also participated in a simulated social interaction observed by a third, untrained informant who completed the same six measures. This design was based on research showing discrepancies between parent and adolescent reports of adolescent social interaction and allowed the researchers to make predictions about specific results that should and should not occur. Using Principal Components Analysis, they found that “all informants' reports loaded positively onto the trait component, informants' reports from different contexts (i.e., parent vs. UUO) loaded onto the context component in opposite directions, and adolescent self-reports loaded onto the perspective component in a direction opposite of the loadings observed from the two observer informants (i.e., parent and UUO).” Interestingly, patterns of reports by source tended to evidence similar ranks across domains (e.g., parent > teacher; youth < parent).

### Measurement invariance

De Los Reyes et al. noted that studies of measurement invariance attempt to determine whether irrelevant conditions influence the function of measurement devices. These irrelevant conditions should not contain variance related to understanding measurement in the domain is being measured; the author' example of irrelevant conditions was cultural/racial background during the measurement of intelligence. De Los Reyes et al. apply this reasoning in the area of youth mental health and the well-known problems of informant discrepancies where reports about a child's social, emotional, and behavioral problems often evidence differences as assessed by the child, parent, teacher, and other professionals. In the authors' view, the key is to identify sources of both common and unique variance in informants' reports, and they illustrate both problems and opportunities to improve youth measurement using this approach. Their key takeaway is that “Efforts to distinguish between domain-relevant and domain-irrelevant measurement conditions should precede use of measurement invariance techniques.”

### Working Alliance Inventory psychometric properties

Paap et al. examined the psychometric properties of the Working Alliance Inventory (WAI) *via* a review of 66 studies published during 1989–2021. The WAI is the most frequently employed measure for studying the working alliance, the connection between client and therapist that has been empirically demonstrated to be related to therapy outcomes. Sample sizes of review studies ranged from 8 to 1,786 participants; mean age ranged from 6 to 98 years; and WAI studies were conducted in 23 countries and 16 languages. Using COSMIN criteria, they found that evidence for measurement properties was lacking in most studies. This includes a lack of evidence for content validity, factor structure, and reliability estimates; Paap et al. also reported conflicting evidence for divergent (discriminant) validity. The authors concluded that further research is needed regarding the theoretical framework underlying the measurement of the working alliance.

### MIMIC model for cognitive neuroscience

Rosen et al. noted that while cognitive neuroscience has provided methods that enhance detection of signal-to-noise ratio from neuroimaging data, problems remain in summarizing behavioral data using aggregated scores, and item response theory (IRT). Rosen et al. also observed that differential item functioning (DIF) can be present with cognitive neuroscience data and that techniques such as the Multiple Indicator Multiple Cause (MIMIC) model can identify and cope with these issues. Previous research has applied the MIMIC model to explore brain-behavior relationships (Kievit et al., 2011, 2012), allowing researchers to model an individual's cognitive ability onto their brain volume. Similarly, this research, using simulations and an empirical study, demonstrated how measurement techniques used to describe brain-behavior relationships can improve statistical power.

## Author contributions

The author confirms being the sole contributor of this work and has approved it for publication.
